# A surgical technique for lesions involving the ultra-high-riding carotid sheath based on the anatomical principles of the prevertebral fascia: cadaveric dissection and case illustrations

**DOI:** 10.3389/fsurg.2026.1781651

**Published:** 2026-03-19

**Authors:** Zhong Liu, Jie Liu

**Affiliations:** 1 Department of Neurosurgery, Zhongshan Hospital of Xiamen University, School of Medicine, Xiamen University, Xiamen, Fujian, China; 2 Department of Neurosurgery, The People’s Hospital of Hezhou, Hezhou, Guangxi, China

**Keywords:** anatomical principles, craniocervical junction, extreme lateral approach, prevertebral fascia, ultra-high-riding carotid sheath

## Abstract

**Background:**

The classic extreme lateral approach (ELA) requires posterior retraction of the sternocleidomastoid muscle (SCM) to expose the ultra-high-riding carotid sheath (UHRCS), which is precisely defined as the segment of the carotid sheath (CS) extending from the site where the sigmoid sinus transitions into the jugular bulb (JB) and exits the skull base to the transverse process of the C2 vertebra. Doing so easily injures the parotid gland (PG) and carotid sheath (CS) and causes significant traction on the internal jugular vein (IJV) and accessory nerve. Thus, this study aimed to describe a novel surgical technique based on the anatomical principle of the prevertebral fascia (PVF) for treating UHRCS-involving lesions.

**Methods:**

Five cadaveric head specimens (10 sides) were used for anatomical research. A standardized ELA position was established, and soft-tissue dissection was modified. The SCM was retracted anteriorly via the natural space of the PVF to expose the UHRCS through the posterior surgical corridor. Based on this, 11 UHRCS-involving lesions were safely resected.

**Results:**

This technique effectively avoided IJV obstruction and reduced traction on the IJV and accessory nerve during UHRCS exposure. Standardized positioning better utilizes PVF anatomical characteristics and the triangular corridor formed by the mastoid tip, C1 transverse process, and occipital condyle, significantly improving exposure efficiency and surgical safety. No PG/CS injuries or new neurological dysfunctions occurred in the 11 surgeries.

**Conclusion:**

The PVF anatomy-based ELA, via standardized positioning, optimized SCM retraction, and key anatomical landmark-guided corridor construction, avoids classic ELA risks and reduces the learning curve. Thus, it provides a safer and more efficient surgical option for UHRCS-involving lesions.

## 1 Introduction

Previous studies have not specifically segmented the carotid sheath (CS) (1, 2). Researchers generally define high cervical carotid artery stenosis as a condition where plaque at the end of the internal carotid artery (ICA) is located at or above the axis (C2), as observed on computed tomography angiography (CTA) or digital subtraction angiography (DSA) (1, 3). The aim of this study is to describe a novel, prevertebral fascia (PVF)-based extreme lateral approach (ELA) that optimizes exposure of ultra-high-riding carotid sheath (UHRCS)-involving lesions, reduces surgical risks, improves procedural safety, and clarifies the anatomical definition of UHRCS.

Before elaborating on the surgical technique, a precise anatomical definition of the UHRCS is first proposed and clarified in detail: the UHRCS refers specifically to the segment of the CS that spans from the skull base exit of the jugular bulb (JB)—where the sigmoid sinus terminates and forms the JB—to the transverse process of the C2 vertebra. Notably, this definition is based on the following four considerations: ① Although the UHRCS is anatomically continuous with the JB above and the CS below (4), there are essential differences in neurosurgical procedures—exposure of the JB often requires grinding of the mastoid process or occipital condyle (OC), exposure of the CS below the axis can adopt a soft tissue dissection method similar to that of carotid endarterectomy (CEA), while the UHRCS has unique technical points for exposure (2, 3, 5, 6); ② Confusing the anatomical concepts of the UHRCS, the JB above it, and the CS below it often makes it difficult for surgeons to grasp key points during surgical training. Only after clarifying these relationships can one fully understand the operational essentials for exposing these three regions: ③ Lesions in the jugular foramen (JF) often involve the JB, UHRCS, or CS simultaneously (7–9). As the anatomical “bridge” between the other two, the UHRCS requires clear differentiation in anatomical structure, exposure methods, and operational details. This is needed to ensure adequate understanding of imaging findings, formulation of detailed preoperative plans, and ultimately “tailored” treatment; ④ Additionally, although the hypoglossal canal (HC) is not located within the CS, the hypoglossal nerve also merges into the CS, and its surgical operational points share many commonalities with those of the UHRCS region (10, 11). Therefore, from multidimensional perspectives in medical education, scientific research, and clinical practice, we have precisely defined the UHRCS and conducted specialized research on it. These four core anatomical and surgical criteria for UHRCS classification serve as the fundamental basis for clinical decision-making in the management of UHRCS-involving lesions, directly guiding patient selection, surgical pathway design, and intraoperative exposure strategy formulation, and laying a critical anatomical foundation for the development of the PVF-based ELA described in this study.

The ELA is a classic surgical technique for managing JF lesions, with extensive research conducted by scholars such as Fukushima and Sekhar (12–16). Classic ELA requires retracting the sternocleidomastoid muscle (SCM) posteriorly, a process that easily injures the accessory nerve; meanwhile, the internal jugular vein (IJV) in this region is located at the outermost side of the CS, and most space-occupying lesions here originate from nerves (7). Thus, when retracting the SCM posteriorly, the IJV becomes a significant obstacle to the operation. Especially when the IJV still maintains normal drainage function, excessive traction increases the risk of complications such as rupture and bleeding, thrombosis, and increased intracranial pressure (17). While the ELA is widely used for skull base and high cervical lesions, standardized, safe surgical strategies specifically targeting UHRCS-involving lesions remain lacking. Unlike conventional ELA or anterolateral approaches (7, 18), our technique leverages the natural space of the PVF to retract the SCM anteriorly, creating a posterior corridor that avoids direct manipulation of critical neurovascular structures (e.g., IJV, accessory nerve). This approach has not been specifically reported for UHRCS lesions in prior studies.

Building on our team's prior work on PVF-based soft-tissue dissection in the CCJ (17), we have proposed a minor technical refinement to the ELA by fully utilizing the PVF's natural space. This refinement adheres to CCJ soft-tissue anatomical principles and aligns with the growth patterns of JF and UHRCS tumors. It effectively avoids accessory nerve and IJV traction, reduces parotid gland (PG) and CS injury risk, and enables more efficient exposure of JF and UHRCS lesions, thereby enhancing surgical safety and efficiency.

## 2 Materials and methods

### 2.1 Overview of cadaveric preparation and dissection

Five adult cadaveric heads (10 sides in total) were used for anatomical observation in this study. To fully utilize the specimens, two cadaveric specimens were reused, and this has been clearly stated in the captions of Figures. The specimen preparation method referred to the previous research of our team (17, 19), which is briefly described as follows: specimens were first perfused with formalin solution for preservation. The surgical position and basic operational steps of the ELA were referenced from previous studies with slight modifications: The specimens were fixed on a self-made head frame, placed in a 3/4 lateral prone position, and the neck skin was pulled with rubber bands to moderately stretch and flex the neck; the head was rotated 30° toward the contralateral side of the lesion, so that the mastoid process was at the highest point, to fully open the atlantooccipital joint (AOJ) and expand the operation space. A postauricular “C"-shaped incision was chosen. The suboccipital muscles were dissected layer by layer until the suboccipital triangle (SOT) was exposed, and all subsequent operations were performed under a microscope (17, 19).

### 2.2 Selection criteria for clinical cases and surgical indications

This was a retrospective, single-center study. All cases included in this study were consecutive patients admitted to the authors' institution from January 2022 to June 2025. This was done to reduce selection bias and ensure the sample's representativeness. 2 authors of this article performed the surgeries. Both surgeons had rich experience in CCJ surgeries and received systematic anatomical training. They had a thorough understanding of both the standardized technique of the classic ELA and the anatomical principles and clinical application of the PVF, ensuring consistent technical implementation.

#### 2.2.1 Surgical indications

All cases involved CCJ lesions, including arteriovenous fistulas, schwannomas, and glomus jugulare tumors (GJT), with the central part of the lesion or its involved range located in the UHRCS.

#### 2.2.2 Exclusion criteria

Patients were excluded if they had a history of neck surgery or radiotherapy (which might cause fascial layer adhesion or anatomical structure deformation), suffered from severe underlying diseases (such as coagulation disorders), could not tolerate surgery, or had malignant tumors that extensively invaded surrounding tissues (which might damage the integrity of the PVF).

## 3 Results

### 3.1 Comprehensive anatomical and surgical investigation

#### 3.1.1 Position

**Figure 1:**
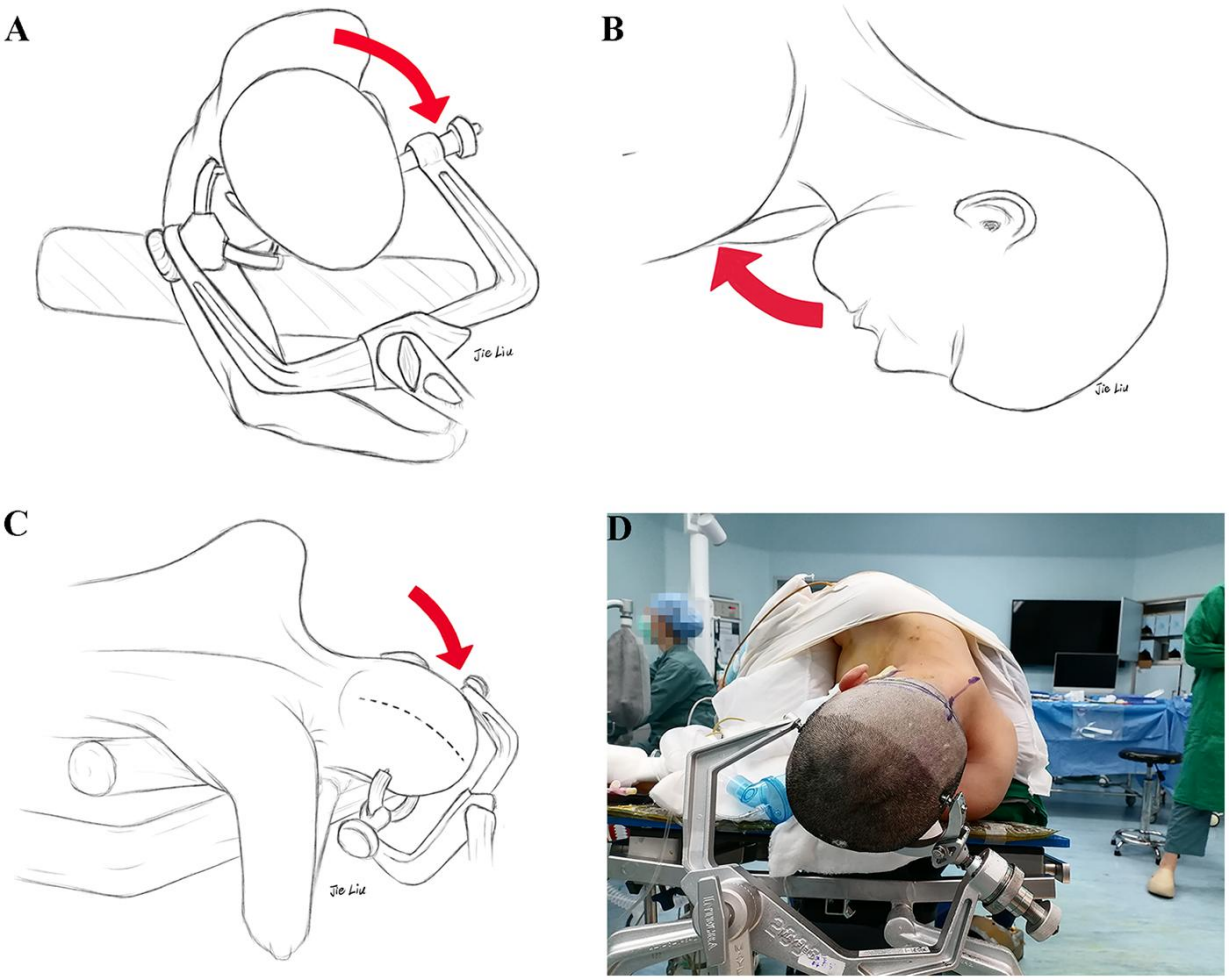
Schematic diagrams **(A–C)** and intraoperative photograph **(D)** showing the position of the ELA. The head is fixed in a three-pin head frame, and after adjustment through three key steps, the AOJ is fully opened and the UHRCS is shifted posteriorly. **(A)** The head is rotated approximately 30∼45° toward the healthy side. **(B)** The neck is flexed so that the distance between the mandible and the manubrium sterni is about 1∼2 horizontal fingers. **(C)** The head is lowered approximately 20° to place the mastoid process at the highest point of the surgical field. **(D)** Overall view of the actual intraoperative body position. ELA, extreme lateral approach; AOJ, atlantooccipital joint; UHRCS, ultra-high-riding carotid sheath.

**Figure 2:**
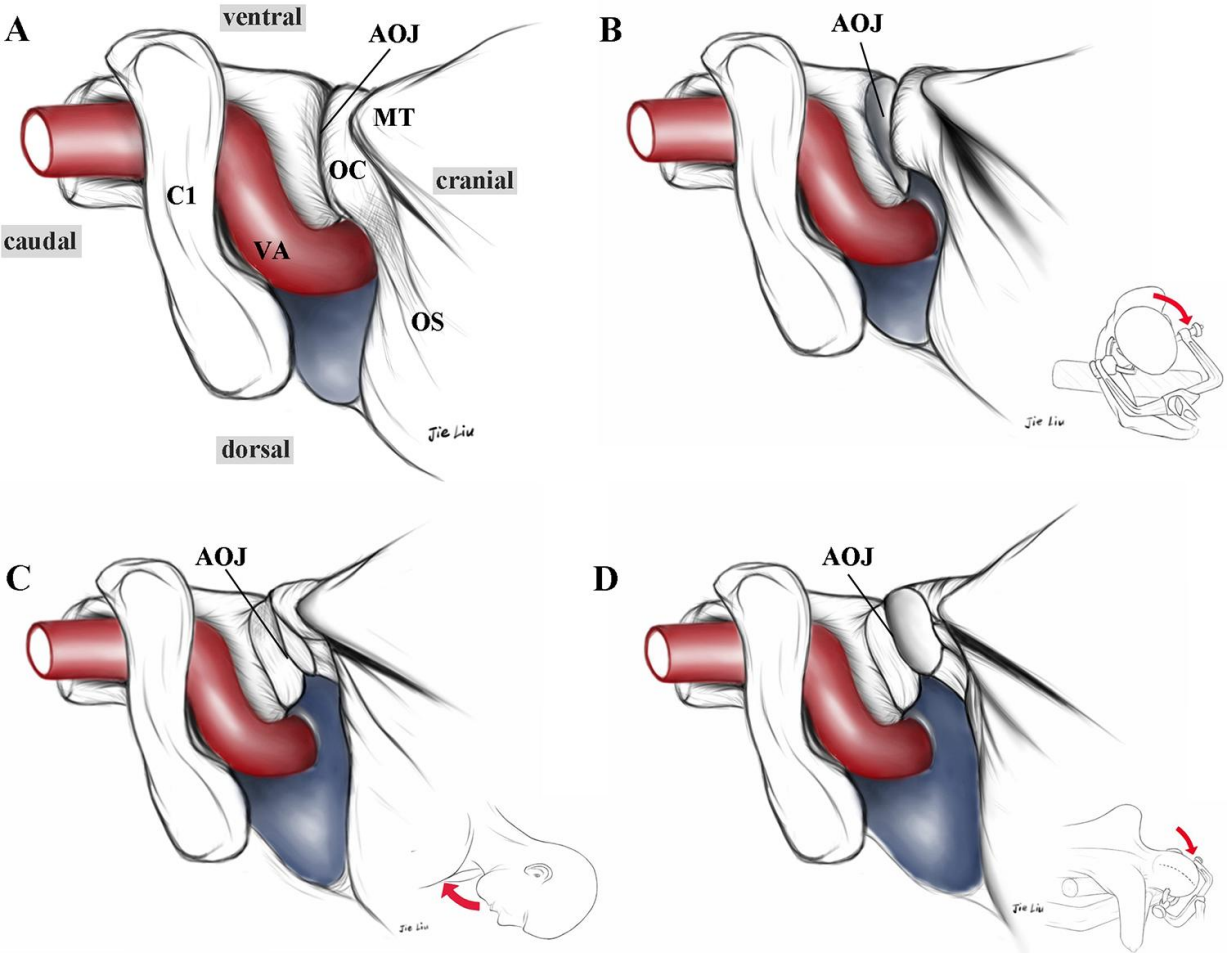
Schematic diagrams showing the conventional lateral position for exposing the CCJ **(A)**, and the standardized adjusted lateral prone position for the ELA **(B–D)**. By adjusting three key steps—neck rotation **(B)**, neck flexion **(C)**, and head drooping **(D)**—the AOJ is fully opened, significantly increasing the operating space in the CCJ (shaded area). The insets in the lower right corner of **(B–D)** show the overall view of the head position. CCJ, craniocervical junction; ELA, extreme lateral approach; AOJ, atlantooccipital joint; C1, atlas; VA, vertebral artery; OC, occipital condyle; MT, mastoid tip; OS, occipital squama.

The 3/4 lateral prone position, also known as the Fukushima or Park Bench position, was adopted, with the axillary pillow properly elevated (Figure 1A). To ensure sufficient surgical operating space, three key steps were required for head positioning: first, rotate the head to the healthy side by approximately 30∼45° until the alae nasi were parallel to the ground (Figures 1A,B). Second, the neck was flexed until the distance between the mandible and the manubrium sterni was close to 1∼2 horizontal fingers (Figures 1B,C). Finally, we let the top of the head droop by approximately 20°, so that the tip of the mastoid process was at the highest point of the surgical field (Figures 1C,D, 2D).

Through this standardized positional adjustment, the surgical corridor of the ELA can be significantly expanded (Figure 2). The operating space of this approach is mainly formed by three anatomical landmarks: the mastoid tip (MT), the C1 transverse process (C1TP), and the OC. Notably, this positional adjustment offers four advantages: 1. Cervical rotation displaces the CS situated beneath the SCM posteriorly, thereby enhancing the feasibility of surgical maneuvers via the retromastoid corridor for the ELA, which is guided by the anatomical principles of the PVF; 2. Effectively expanding the operating space of the ELA formed by the MT, C1TP, and OC; 3. Widening the interval between the atlas and foramen magnum, thereby providing convenience for resecting the C1TP and displaced VA; 4. Achieving more adequate exposure of the AOJ, which facilitates grinding the posterior 1/3 of the OC to expose the HC.

**Figure 3:**
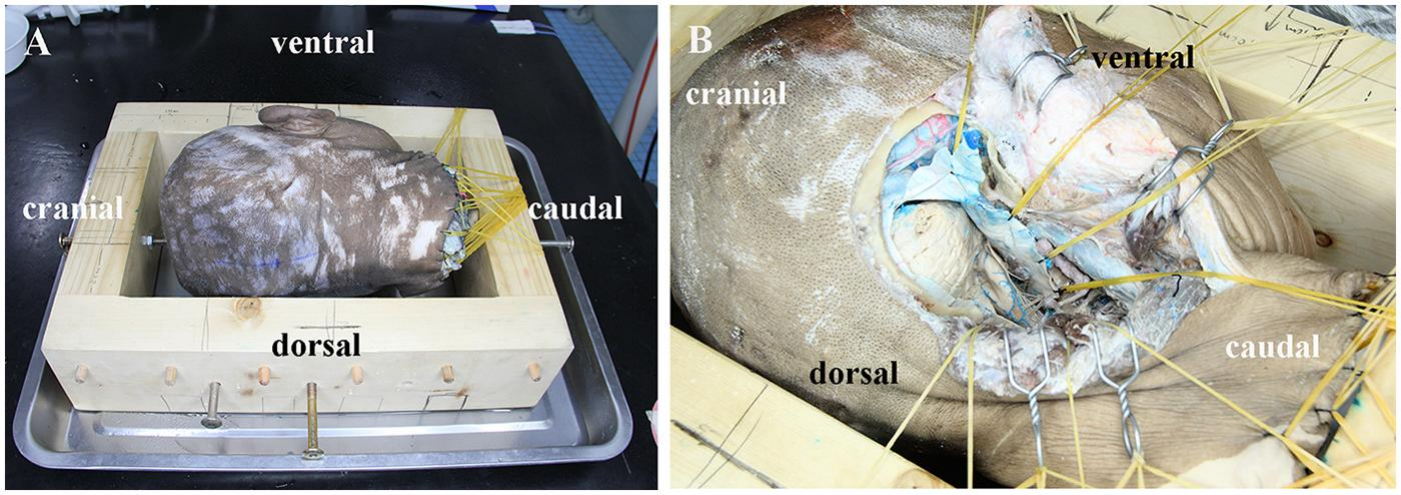
Application of the self-made anatomical head frame in the ELA. **(A)** The self-made anatomical head frame is equipped with screws on all four sides to fix the skull, achieving stable fixation; additionally, multi-directional free traction with rubber bands can assist in opening the AOJ and simulating the head position requirements in actual surgery. **(B)** Cadaveric head specimen showing the overall view of the exposure field for the ELA achieved by applying the anatomical principles of the PVF. ELA, extreme lateral approach; AOJ, atlantooccipital joint; PVF, prevertebral fascia.

To more accurately simulate intraoperative positioning requirements on specimens, we designed a new anatomical head frame and have applied for a patent (Figure 3). This head frame has four core advantages: first, its manufacturing process is simple and the cost is low; second, the fixation operation is convenient, enabling stable fixation of cadaveric head specimens; third, the traction function is flexible, allowing all-round traction adjustment of skin flaps and muscle flaps; finally, relying on the above three advantages, this head frame can and efficiently simulate the clinical intraoperative surgical position (especially the lateral prone position), which is also the most important difference and advantage compared with other anatomical head frames.

#### 3.1.2 Incision and flap

Due to the close anatomical relationships among the UHRCS, JF, JB, and CS, the skin flap design must be carefully tailored to the specific conditions of the lesion to clarify the surgical goals. When only epidural structures such as the UHRCS, JF, and JB need to be exposed, a small “C"-shaped incision is sufficient; if the lesion involves both subdural and epidural regions, craniotomy of the occipital squama (OS) must be considered, and the posterior edge of the “C"-shaped incision should be closer to the midline in this case. If the patient has IJV occlusion or severe stenosis, the compensatory status of the external jugular vein (EJV) should also be evaluated, and the skin flap design must avoid damaging the compensatory EJV as much as possible. Detailed diagrams of the specific incision design have been elaborated in previous studies of our team (17, 19).

**Figure 4:**
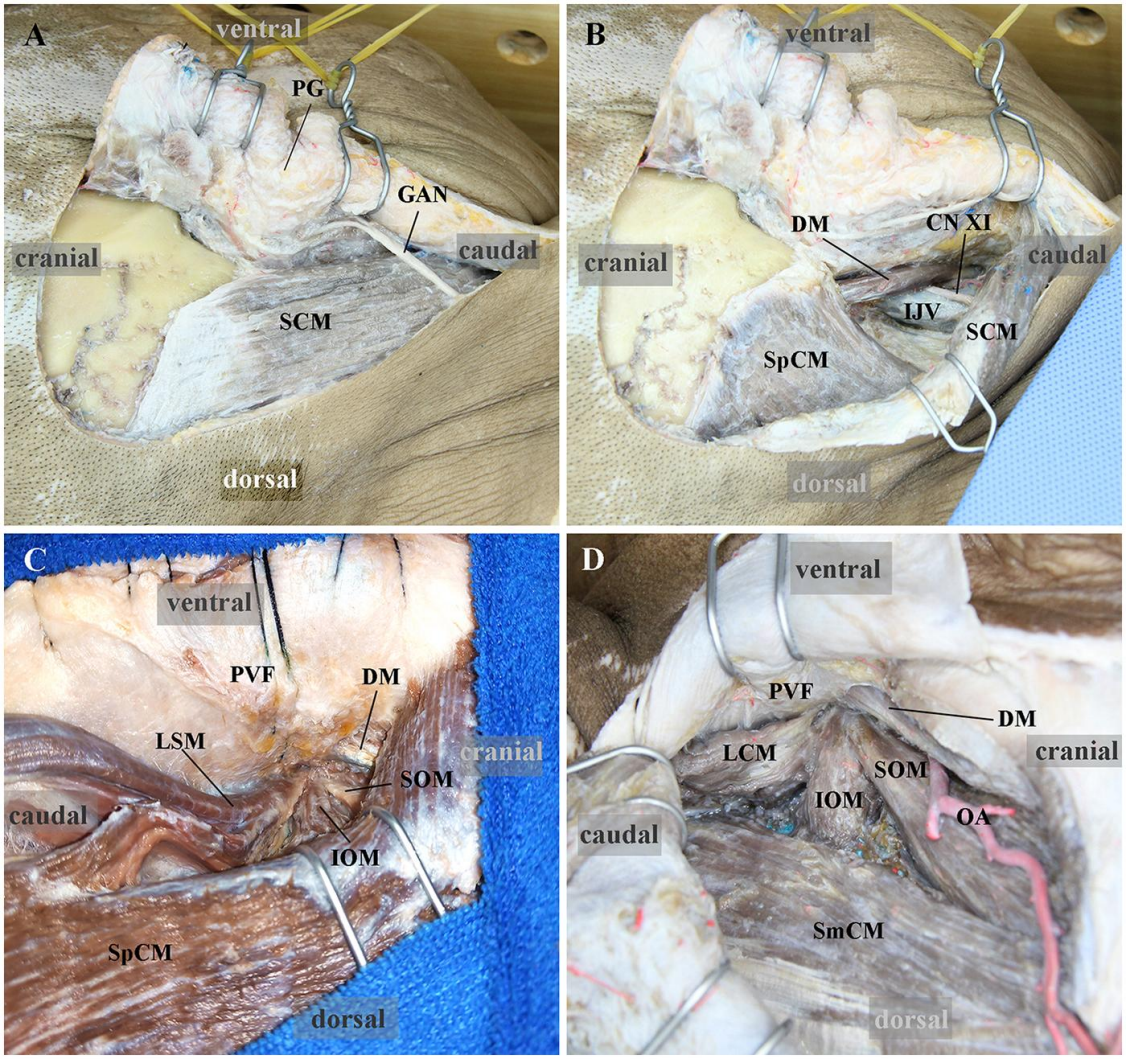
Skin flaps for the classic ELA **(A,B)** and the ELA performed based on the anatomical principles of the PVF **(C,D)**. **(A)** When retracting the skin flap in the classic ELA, the PG is easily exposed, and the GAN also causes obvious obstruction. **(B)** When retracting the SCM posteriorly, there is traction on the accessory nerve and obstruction of the deep PCN by the IJV. **(C)** The SCM and PVF are retracted anteriorly together, thereby avoiding exposure of the PG and traction on the accessory nerve. **(D)** In the far lateral approach, the SCM can also be retracted anteriorly, along with the PVF, to locate the PVF in another specimen. ELA, extreme lateral approach; PG, parotid gland; GAN, great auricular nerve; SCM, sternocleidomastoid muscle; IJV, internal jugular vein; PVF, prevertebral fascia; DM, digastric muscle; SpCM, splenius capitis muscle; CN XI, accessory nerve; LSM, levator scapulae muscle; SOM, superior oblique muscle; IOM, inferior oblique muscle; SeCM, semispinalis capitis muscle; LCM, longissimus capitis muscle; OA, occipital artery; PCN, posterior cranial nerves.

One of the key steps of the ELA, which is performed based on the anatomical principles of the PVF, is to retract the skin flap and SCM anteriorly as a single layer and then dissect along the medial surface of the SCM to identify the PVF. After locating the PVF, detach the splenius capitis muscle (SpCM) from the mastoid process and retract it posteriorly; dissect the ongissimus capitis muscle (LCM), retract it posteroinferiorly, resect the superior obliquus muscle (SOM) and rectus capitis lateralis muscle (RCLM), and then detach the levator scapulae muscle (LSM) from the C1TP before retracting it posteroinferiorly. Throughout the muscle flap dissection, the operation must be performed consistently posterior to the PVF to avoid inadvertent entry into the CS (Figure 4).

**Figure 5:**
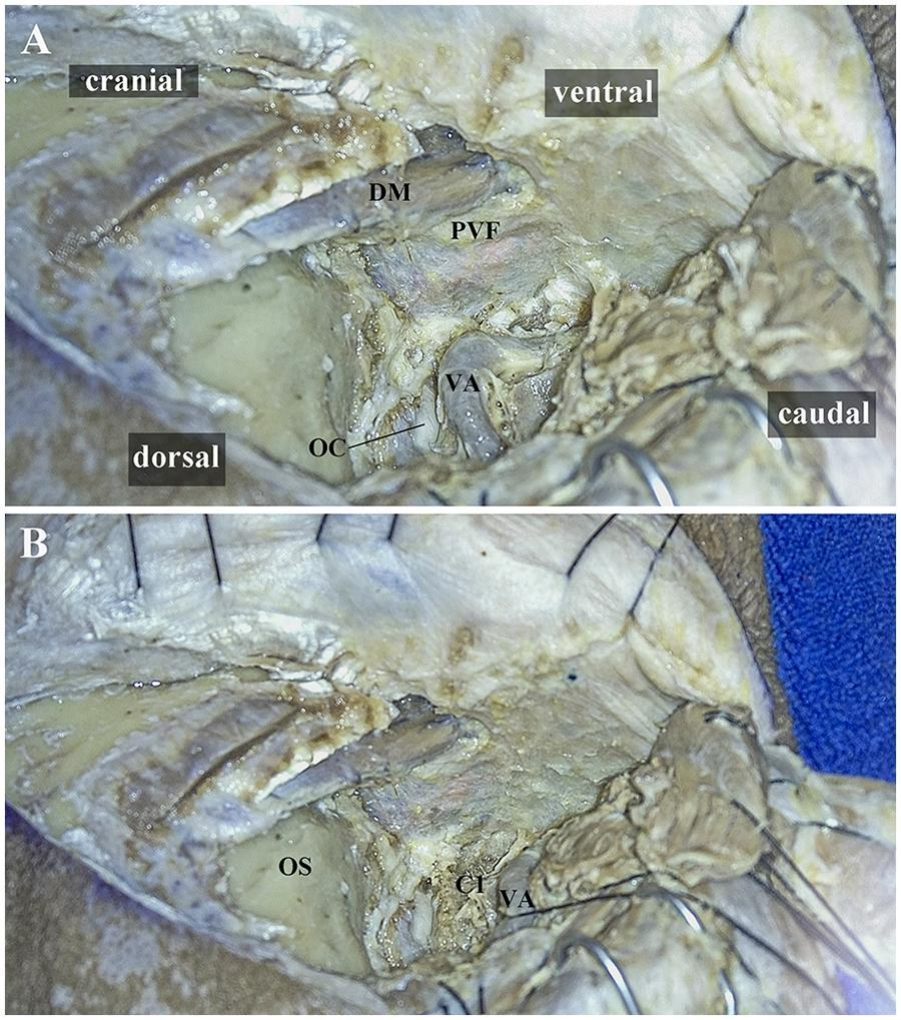
Cadaveric head dissection of the right-sided ELA performed based on the anatomical principles of the PVF. **(A)** During exposure of the UHRCS using PVF anatomical principles, if the C1TP significantly obstructs the C1 transverse foramen, it can be opened. **(B)** The VA can be freed and retracted posteroinferiorly to expand the exposure range further. ELA, extreme lateral approach; UHRCS, ultra-high-riding carotid sheath; PVF, prevertebral fascia; C1, atlas; C1 transverse process, C1TP; DM, digastric muscle; VA, vertebral artery; OC,occipital condyle; OS, occipital squama. Adapted with permission from (17), licensed under CC BY.

**Figure 6:**
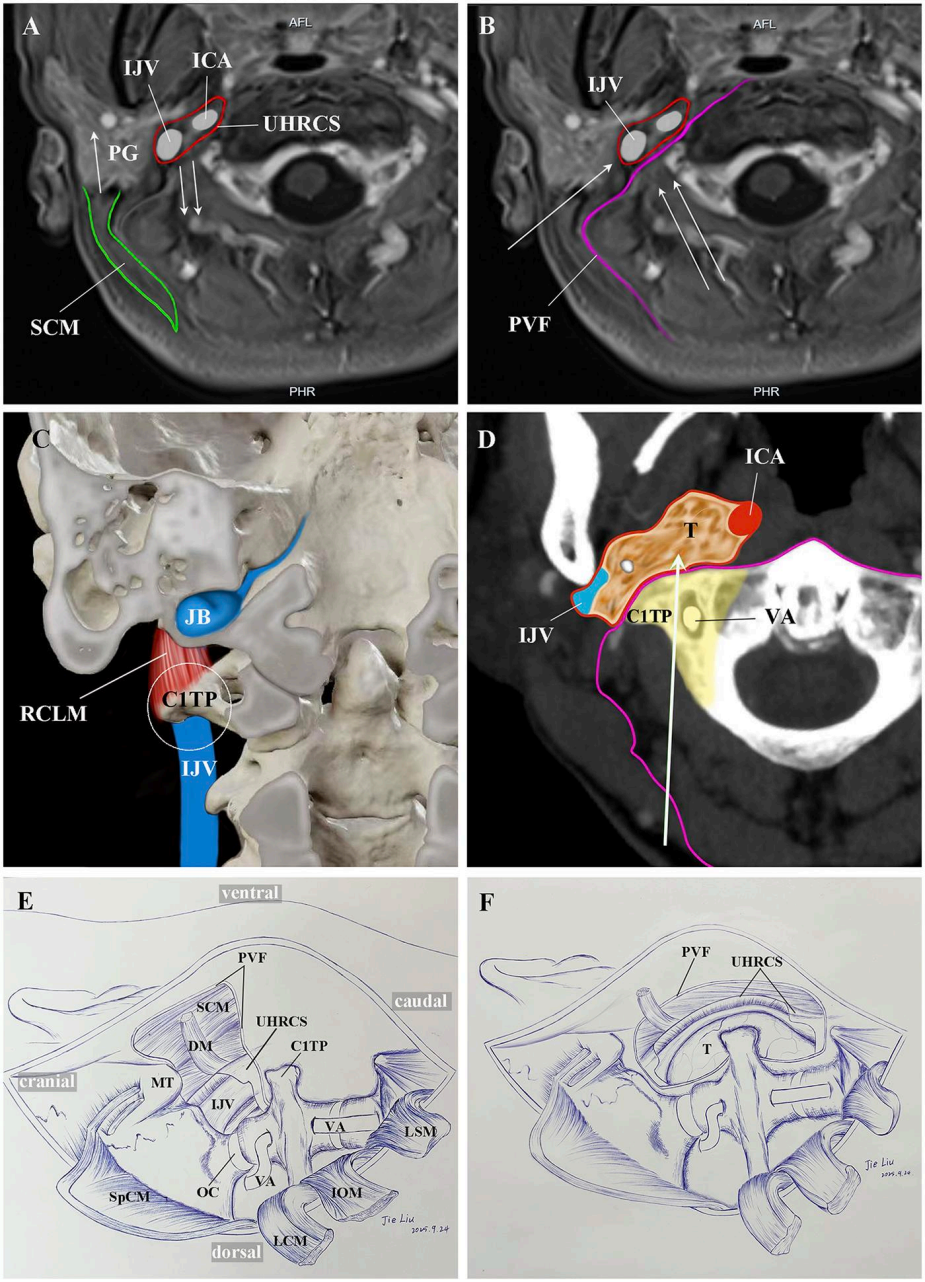
Schematic diagram of the clinical application of the ELA based on the anatomical principles of the PVF. Panels **A** and **B** show the magnetic resonance (MR) axial images of the UHRCS. **(A)** In the ELA based on the anatomical principles of the PVF, rotation of the head and neck can make the SCM (green) and UHRCS (red) move toward each other: the SCM rotates anteriorly (single arrow), and the UHRCS rotates relatively posteriorly (double arrow). This is more conducive to utilizing the anatomical principles of the PVF (pink) to expose the UHRCS and simplify intraoperative operations. **(B)** The surgical path of the classic ELA (single arrow) is prone to injury to the PG and UHRCS; in contrast, the proposed technique (double arrow) fully utilizes the anatomical principles of the PVF (pink), avoiding injury to the PG and UHRCS. **(C)** A three-dimensional coronal schematic diagram of the left JF, showing the relationship between the UHRCS, JB, C1TP, and surrounding bones. The obstructive effects of the C1TP and RCLM (marked by a circle) on the exposure of the UHRCS are evident. **(D)** Axial CTA image of the UHRCS, showing the obstruction of the lesion by the C1TP in the ELA performed based on the anatomical principles of the PVF; the C1TP can be drilled intraoperatively to expand the exposure range. It can be observed that lesions in the UHRCS often displace the IJV laterally and push the ICA medially, an anatomical principle that the ELA based on the anatomical principles of the PVF fully utilizes. Panels **(E)** and **(F)** are schematic diagrams of the normal anatomical approach of the ELA based on the anatomical principles of the PVF **(E)** and the approach when a lesion exists in the UHRCS **(F)**, respectively. ELA, extreme lateral approach; PVF, prevertebral fascia; UHRCS, ultra-high-riding carotid sheath; SCM, sternocleidomastoid muscle; ELA, extreme lateral approach; PG, parotid gland; JB, jugular bulb; C1TP, C1 transverse process; CTA, computed tomography angiography; IJV, internal jugular vein; ICA, internal carotid artery; RCLM, rectus capitis lateralis muscle; T, tumor; VA, vertebral artery; MT, mastoid tip; OC, occipital condyle; DM, digastric muscle; LSM, levator scapulae muscle; SOM, superior oblique muscle; IOM, inferior oblique muscle; SeCM, semispinalis capitis muscle; SpCM, splenius capitis muscle; LCM, longissimus capitis muscle.

For complications with subdural space-occupying lesions, the semispinalis capitis muscle (SeCM), rectus capitis posterior major muscle (RCPMaM), and rectus capitis posterior minor muscle (RCPMiM) need to be additionally retracted posteroinferiorly; if the lesion within the CS is located deeply and the C1TP causes significant obstruction, the C1TP can be drilled, the V3 segment of the VA can be freed entirely, transposed, and retracted posteromedially and inferiorly. The C1TP can be thoroughly drilled to expand the surgical field (Figures 5, 6).

### 3.2 Relationship between the PVF and UHRCS

**Figure 7:**
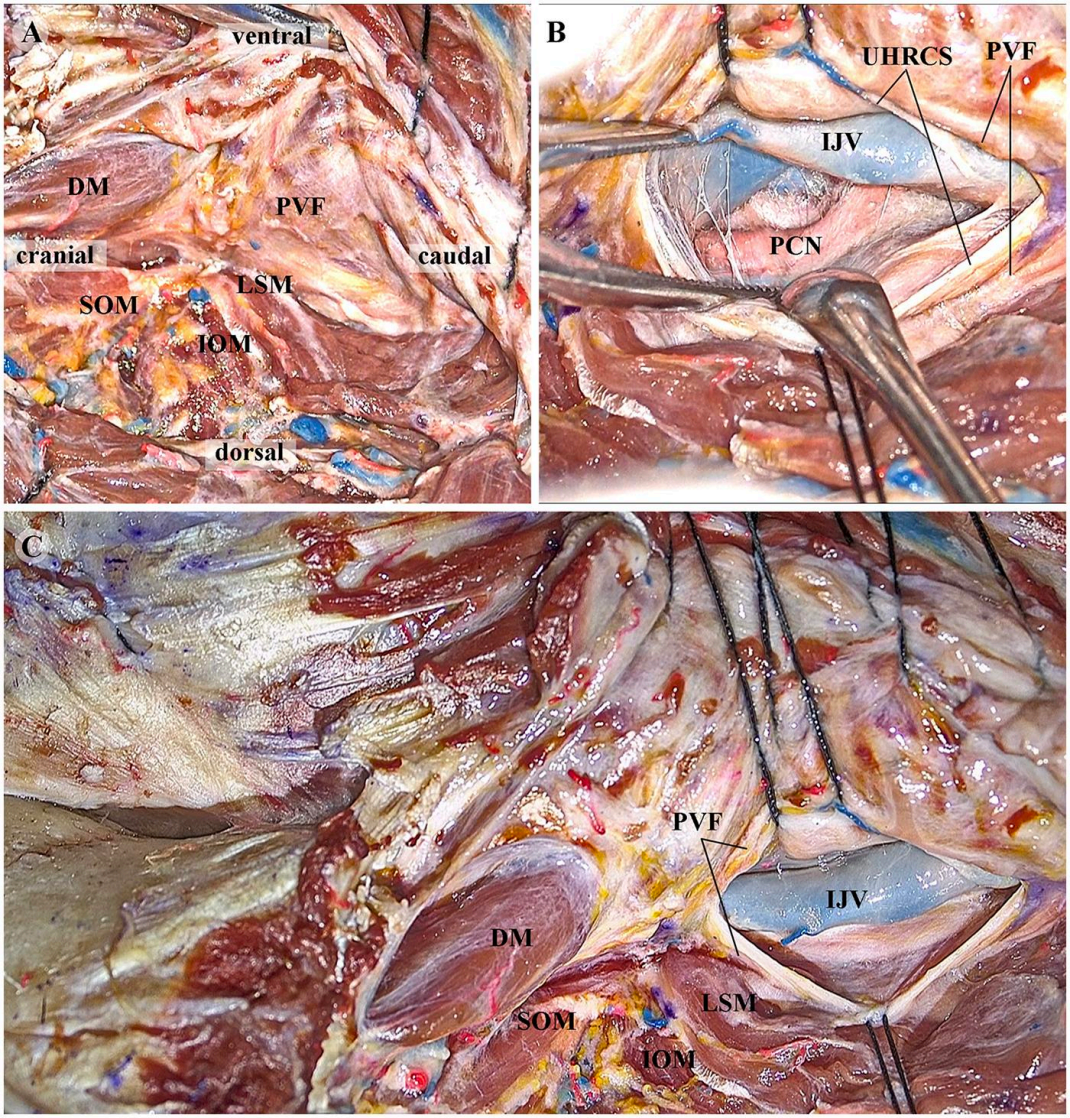
Cadaveric head specimens showing exposure of the UHRCS via the ELA based on the anatomical principles of the PVF. **(A)** In the ELA, the lateral suboccipital muscles are dissected in layers, with the operation consistently performed along the natural space between the PVF and the muscle group. **(B)** The PVF and UHRCS are incised to expose the internal jugular vein (IJV) and the PCN. **(C)** Gross view after exposure of the UHRCS. UHRCS, ultra-high-riding carotid sheath. ELA, extreme lateral approach; PVF, prevertebral fascia; IJV, internal jugular vein; PCN, posterior cranial nerves; DM, digastric muscle; LSM, levator scapulae muscle; SOM, superior oblique muscle; IOM, inferior oblique muscle. *To maximize the use of the specimen, the same specimen was used in this figure as in* Figure 2 *of our prior research* (17).

Except for the posterior belly of the digastric muscle (DM) passing through the PVF, the PVF completely separates the posterior occipital muscles from the CS. Therefore, during the dissection of the posterior occipital muscles, the CS will not be exposed or injured as long as the PVF remains intact. Conversely, after incising the PVF and the CS, the IJV, posterior cranial nerves (PCN), and internal carotid artery (ICA) can be smoothly exposed from the posterior side (Figure 7).

The contents of the CS are arranged as follows: the IJV is posterolateral, the PCNs are in the middle, and the ICA is anteromedial (Figure 7B, Supplementary Video S1). Most space-occupying lesions within the UHRCS originate from nerves (e.g., schwannomas, paragangliomas). During the development of these lesions, they usually expand around the PGN as the center, pushing the IJV laterally and the ICA anteromedially.

This ELA based on the anatomical principles of the PVF is more in line with the lesions' inherent growth pattern. After opening the PVF and CS, the surgeon can directly access the tumor without retracting the IJV, thereby improving exposure efficiency and ensuring surgical safety.

### 3.3 Surgical results

From January 2022 to June 2025, we performed surgeries for 11 cases of CCJ lesions involving the UHRCS using the ELA based on the anatomical principles of the PVF (Table 1). None of the 11 clinical cases included in this study overlapped with those reported in our previous publication (17), as the current cohort consists of consecutive patients enrolled after the completion of the prior study. All 11 lesions were tumors, and all achieved radiologically complete resection. Two cases developed subcutaneous effusion and fully recovered after one week of pressure bandaging, while no new neurological complications were observed in the other cases. Notably, one case of UHRCS schwannoma complicated with arteriovenous fistulas (AVF) also achieved complete occlusion of the shunt. The specific lesion types included: 5 cases of glomus jugulare tumors involving the UHRCS, 3 instances of schwannomas involving the UHRCS and JF, 1 case of a UHRCS schwannoma complicated by AVF, and 2 cases of schwannomas involving the HC and UHRCS.

**Table 1:** Clinical data of 11 cases with lesions involving the ultra-high-riding carotid sheath treated by extreme lateral approach based on the anatomical principle of the prevertebral fascia.

NO	Age/y	Sex	Lesion type	Lesion location	Approach	Incision	CS injury	PO-NOND
1	68	F	GJT	UHRCS, JB	ELA	"C” shaped	None	None
2	59	M	schwannoma, AVF	UHRCS	ELA	"C"-shaped	None	None
3	57	F	schwannoma	UHRCS, JF	ELA	"C"-shaped	None	None
4	52	M	schwannoma	UHRCS, JF	ELA	"C"-shaped	None	None
5	55	F	GJT	UHRCS, JB	ELA	"C"-shaped	None	None
6	61	M	GJT	UHRCS, JB	ELA	"C"-shaped	None	None
7	47	F	schwannoma	UHRCS, HC	ELA	"C"-shaped	None	None
8	58	F	schwannoma	UHRCS, JF	ELA	"C"-shaped	None	None
9	49	F	GJT	UHRCS, JB	ELA	"C"-shaped	None	None
10	63	F	GJT	UHRCS, JB	ELA	"C"-shaped	None	None
11	53	F	schwannoma	UHRCS, HC	ELA	"C"-shaped	None	None

UHRCS, ultra-high-riding carotid sheath; ELA, extreme lateral approach; AVF, arteriovenous fistulas; JF, jugular foramen; JB, jugular bulb; GJT, Glomus jugulare tumor; HC, hypoglossal canal; PO-NOND, postoperative new-onset neurological deficit.

### 3.4 Illustrative cases

#### 3.4.1 Case 1 (patient 2)

**Figure 8:**
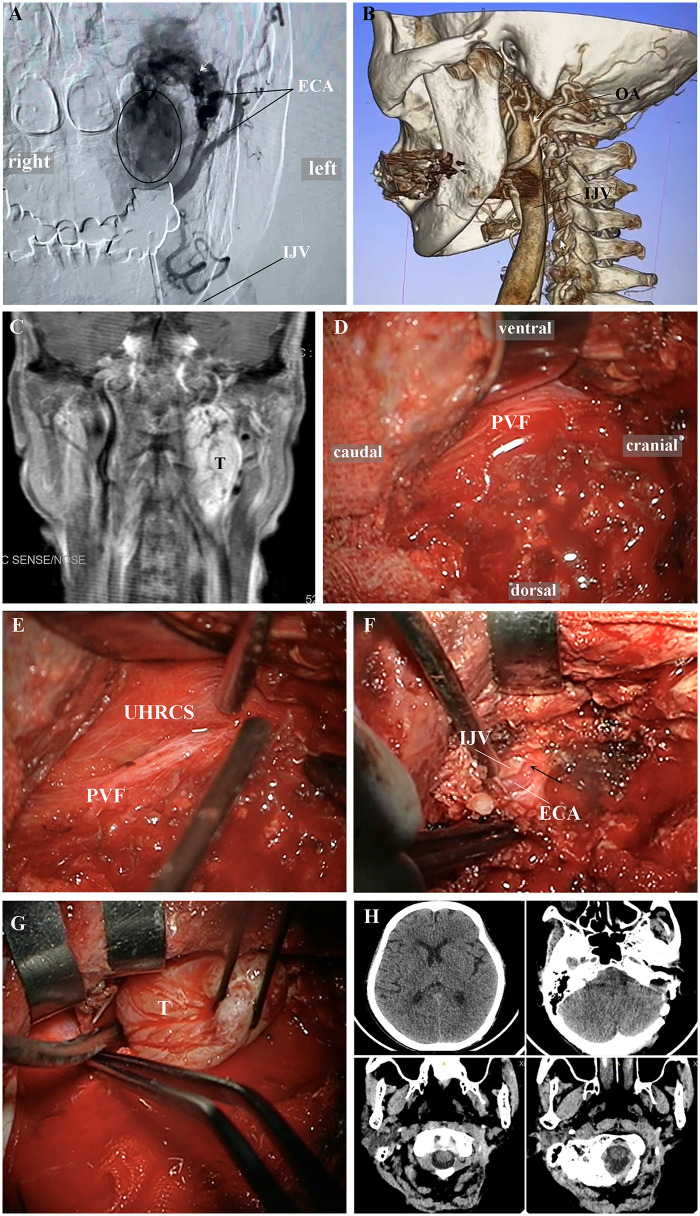
Resection of a left-sided UHRCS space-occupying lesion complicated with AVF via the ELA based on the anatomical principles of the PVF. The key to this surgery is to accurately locate and electrocoagulate the fistula to reduce the tumor's blood supply. **(A)** DSA shows apparent tumor staining and early venous enhancement caused by AVF; the lesion is supplied by branches of the external carotid artery, with the fistula (Arrow) located posterior to the UHRCS and the IJV as the draining vein. **(B)** 3D CTA reconstruction shows the relative relationships among the UHRCS lesion, the fistula (Arrow), and surrounding bony structures and blood vessels. **(C)** MRI shows a space-occupying lesion in the left UHRCS; the fistula provides abundant tumor blood supply. **(D)** A “C"-shaped incision is made, and muscles and soft tissues are dissected according to the anatomical principles of the PVF. **(E)** The PVF is incised to expose the UHRCS. **(F)** The fistula is identified and electrocoagulated; it is located posterior to the UHRCS. **(G)** The tumor is further dissected and resected; it is observed that the tumor's blood supply is significantly reduced after fistula electrocoagulation. **(H)** Postoperative CT scan confirms complete tumor resection. UHRCS, ultra-high-riding carotid sheath; AVF, arteriovenous fistula; ELA, extreme lateral approach; DSA, digital subtraction angiography; OA, occipital artery; IJV, internal jugular vein; ECA, external carotid artery; 3D CTA, three-dimensional computed tomography angiography; MRI, magnetic resonance imaging; PVF, prevertebral fascia; CT, computed tomography; T, tumor.

A 59-year-old male patient was admitted to the hospital due to an intracranial bruit accompanied by neck pain for 3 months. Physical examination revealed a firm, non-tender, and non-mobile mass palpable in the left neck. Imaging studies showed a space-occupying lesion in the UHRCS, with early venous enhancement due to an AVF. The fistula was supplied by the branches of the external carotid artery, located posterior to the UHRCS, and drained into the IJV (Figures 8A–C). Due to the rapid drainage of the fistula directly into the IJV, the risk of interventional embolization for the fistula was relatively high. Therefore, the patient was suitable for surgical resection of the tumor and clipping of the fistula. The left-sided ELA was adopted for the surgery, with a “C"-shaped incision made. Muscles and soft tissues were dissected in accordance with the anatomical principles of the PVF (Figure 8D). The PVF was incised to expose the UHRCS (Figure 8E). After the fistula was identified, it was treated with electrocoagulation (Figure 8F). The tumor was further dissected and resected (Figure 8G). Postoperative computed tomography scan confirmed complete tumor resection (Figure 8H). The patient was discharged on the 12th postoperative day without new-onset neurological complications (Supplementary Video S2).

#### 3.4.2 Case 2 (patient 11)

**Figure 9:**
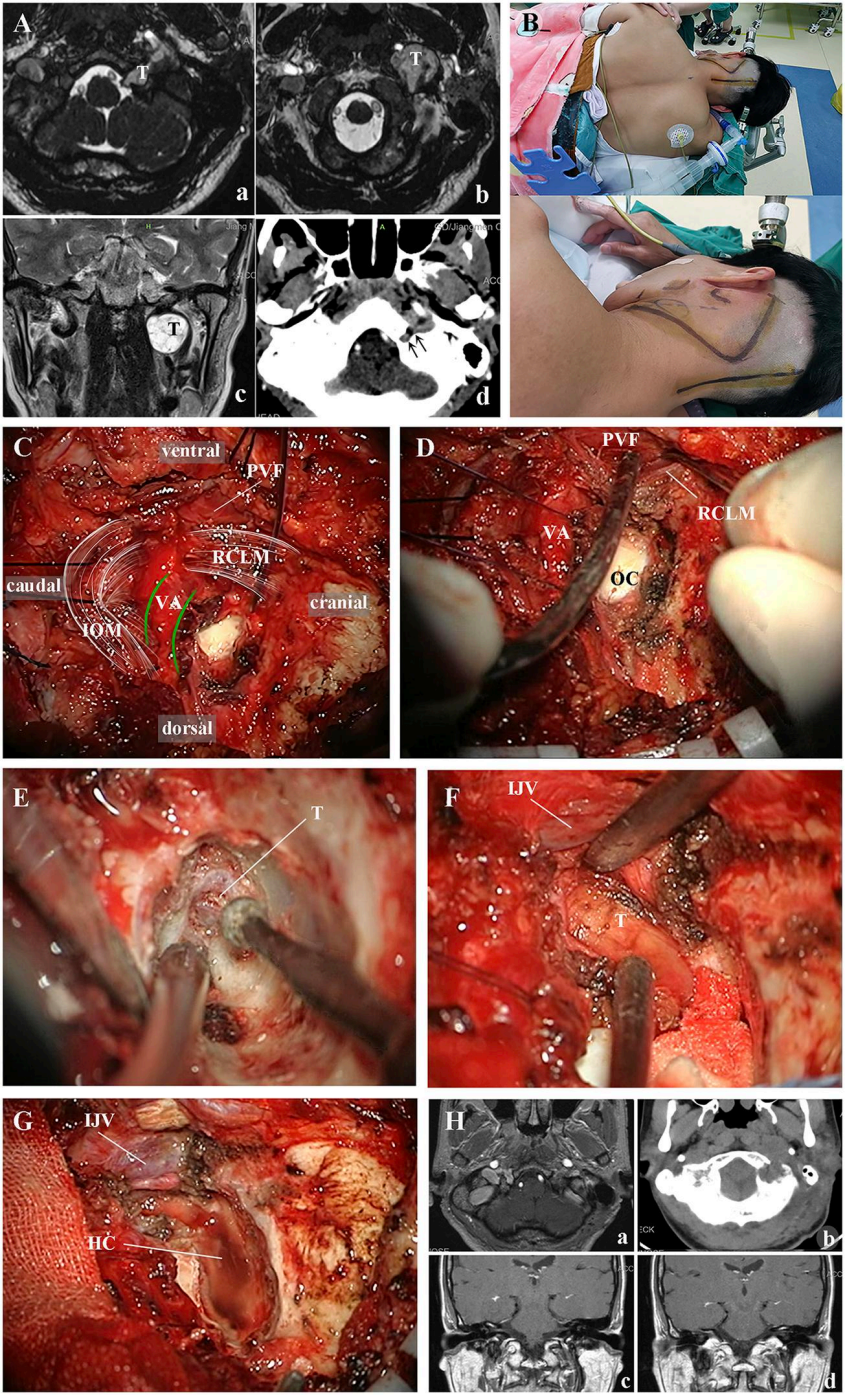
Resection of left-sided HC and UHRCS space-occupying lesion via the ELA based on the anatomical principles of the PVF. **(A)** MRI **(A–C)** clearly identifies the space-occupying lesion in the left HC and UHRCS; CT **(D)** scan shows enlargement of the left HC. **(B)** Intraoperatively, the left lateral prone position and suboccipital lateral “C"-shaped incision are adopted. **(C)** Muscles and soft tissues are dissected according to the anatomical principles of the PVF. The perivertebral venous plexus around the VA is released using the PTRT (due to blurriness in the intraoperative video, white lines mark the course and contour of muscles, and green lines mark the contour of the VA). **(D)** The OC, RCLM, and PVF are exposed. **(E)** The posterior 1/3 of the OC is drilled to expose the tumor in the HC. **(F)** The RCLM is resected, and the PVF is opened to reveal the tumor in the UHRCS; the tumor is seen to push the IJV laterally. **(G)** After tumor resection, the IJV is well protected, eliminating the need for traction on it. **(H)** Postoperative MRI **(A,C,D)** and CT **(B)** scan confirm complete tumor resection. HC, hypoglossal canal; UHRCS, ultra-high-riding carotid sheath; ELA, extreme lateral approach; MRI, magnetic resonance imaging; CT, computed tomography; PVF, prevertebral fascia; PTRT, Posterior atlantooccipital membrane (PAOM) tension release technique; VA: vertebral artery; OC, occipital condyle; RCLM, rectus capitis lateralis muscle; T, tumor; IJV, internal jugular vein.

A 53-year-old female patient visited our hospital due to dysphagia for more than 2 years. Physical examination revealed a firm, non-tender, and non-mobile mass palpable in the left neck. Preoperative magnetic resonance imaging showed a space-occupying lesion in the left HC and UHRCS, consistent with a schwannoma, with enlargement of the left HC (Figure 9A). The surgery was performed via the ELA with a left suboccipital lateral “C"-shaped incision (Figure 9B) to resect the tumor. The perivertebral venous plexus around the VA was released using the PTRT (19) (Figure 9C). The OC was exposed, and the posterior 1/3 of it was drilled (Figure 9D). After exposing the HC, the PVF was incised to fully expose the tumor, which was then completely resected (Figures 9E–G). Postoperative MRI confirmed complete tumor resection (Figure 9H). The patient was successfully discharged on the 14th postoperative day without new-onset neurological complications.

## 4 Discussion

### 4.1 Development history of the ELA and related techniques

The development of the ELA began in 1990, when Sen and Sekhar first proposed this approach and successfully applied it to manage intradural lesions in the upper cervical segment and ventral foramen magnum (12). Subsequent extensive anatomical and clinical studies gradually enhanced its clinical recognition. In 1994, Babu further expanded this technique by proposing the extreme lateral transcondylar approach (13). In 1999, Sekhar's team (with Salas) systematically improved the ELA system and innovatively proposed 6 variants of the approach. This transformed the ELA from a single surgical method into a multi-scenario adaptive technical system (7).

During the same period, Fukushima's ELA research also marked a milestone. In 1987, he proposed the extreme lateral infrajugular transcondylar–transtubercular exposure (ELITE) technique, identifying “grinding the jugular tubercle” as a key step for exposing the middle and lower clivus, the vertebrobasilar junction, and the ventral pontomedullary region, providing core anatomical evidence for the exposure of deep structures (20). Starting from 2010, Fukushima further expanded the scope of the ELITE concept, dividing it into “dorsolateral ELITE” (original ELITE) and “anterolateral ELITE” (combined approach), marking the formal maturation of the ELITE system (21). Among them, the anterolateral ELITE requires retracting the SCM posteriorly, and its procedures include infralabyrinthine mastoidectomy, JB exposure, foramen magnum opening, condyle drilling, jugular tubercle drilling, and high cervical segment exposure. It is particularly suitable for communicating lesions in the CCJ (foramen magnum–JF–high cervical segment) (14, 15, 21).

The exploration and practice of the aforementioned pioneering scholars have provided key technical support and laid a crucial clinical foundation for the surgical field exposure of lesions in the JF, JB, and CS, with milestone significance. However, this region has complex anatomical structures and dense blood vessels/nerves, making related surgical operations extremely difficult and imposing strict requirements on the surgeon's anatomical knowledge and operational precision. This also poses significant challenges for surgeons learning to operate in this area. Our team has long focused on anatomical research and the surgical practice of the CCJ. Addressing the aforementioned clinical difficulties, we further optimized the surgical approach design. This study focuses on the clinical challenge of “lesions involving the UHRCS”. It highlights the ELA based on the anatomical principles of the PVF, aiming to provide a more operable new option for the clinical treatment of such complex lesions.

### 4.2 Anatomical relationship between the JB and UHRCS, and difficulties in surgical learning

A typical feature of JF lesions is their tendency to form intracranial-extracranial communication, often involving the JF, JB, and UHRCS simultaneously (4, 5). These three structures are closely adjacent anatomically and form an essential neurovascular complex in the CCJ. However, in anatomical training and clinical teaching, we found that most trainees have a vague understanding of the anatomical boundaries, anatomical principles, and functional connections of the JF, JB, and UHRCS. This makes it difficult to accurately locate the lesion range from a surgical perspective during image analysis; when formulating surgical strategies, they can only “follow the example” blindly, which easily leads to positioning deviations. As a result, individualized surgical design is hardly achievable, leading to unnecessary exposure and reduced surgical efficacy.

To solve this problem, based on precise anatomical structures and differences in surgical operations, this study redefines the segment of the CS in the high cervical region that is directly adjacent to the JB and has special anatomical morphology and clinical significance—i.e., the CS segment from the axis transverse process to the skull base exit of the JB—as the “UHRCS”. This definition clarifies the anatomical boundaries between the UHRCS and the JB (above) as well as the conventional CS (below), providing clear anatomical markers for clinical diagnosis and treatment and laying a foundation for the precise implementation of subsequent surgical operations.

The exposure of the UHRCS is a recognized technical difficulty in neurosurgery (2, 3). Although many scholars have conducted extensive research on this region, significant obstacles remain in clinical learning. The core problem lies in the confusion of the anatomical relationships among the JB, UHRCS, and conventional CS. If surgeons cannot clarify the spatial positions, functional connections, and differences in surgical exposure methods among these three structures, they will struggle to grasp the key points of exposure in each region, leading to difficulties such as “blind traction” and “structural misjudgment” during surgery.

Currently, the classic surgical method for managing UHRCS lesions is the ELA, whose core procedure involves severing the SCM from the mastoid tip and retracting it posteriorly to expose the lesion (16, 22, 23). However, this classic operation still requires precise anatomical knowledge for safe implementation in practice, further highlighting the importance of clarifying anatomical concepts for surgical learning. Clarifying the clear anatomical boundaries and surgical criteria of the UHRCS optimizes intraoperative anatomical identification and surgical decision-making, effectively reducing the risks of “blind traction” and “structural misjudgment” during the exposure of UHRCS-involving lesions, and lowering the learning curve for surgeons to master the surgical techniques of this complex anatomical region, which is one of the core novelties of this study in anatomical classification and clinical application.

### 4.3 Limitations of the classic ELA in exposing the UHRCS

Although the classic ELA is a commonly used surgical method for treating UHRCS lesions, there are multiple limitations in its exposure method through long-term anatomical learning and clinical practice, as detailed below:

#### 4.3.1 High risk of IJV injury

Most UHRCS lesions originate from nerves; such lesions often push the IJV laterally, significantly increasing venous tension. In the classic approach, to obtain a sufficient surgical field, the IJV (already under high tension) must be retracted, which increases the risk of venous wall injury, thrombosis, intraoperative bleeding, and postoperative venous reflux.

#### 4.3.2 Vulnerability of the compensatory EJV

When space-occupying lesions exist in the UHRCS area, the IJV is often narrowed or even occluded due to compression. At this time, intracranial venous reflux is mainly compensated by the EJV, resulting in prominent dilatation of the EJV. The classic ELA requires retracting the SCM posteriorly, which necessitates separating the skin flap from the SCM—this process easily damages the compensatory EJV. Additionally, the skin flap design must extend forward to the anterior edge of the SCM to meet requirements for surgical field exposure. These designs are highly likely to damage the dilated compensatory EJV, disrupt the intracranial venous reflux pathway, and further cause complications such as postoperative increased intracranial pressure and cerebral edema.

#### 4.3.3 Significant risk of injury to the accessory nerve and PG

Retracting the SCM posteriorly is a key step in the classic ELA. The extracranial segment of the accessory nerve exits the JF, descends into the posterior cervical triangle, then enters the deep surface of the SCM, and finally reaches the deep surface of the TM. When the inferior pole of the lesion is low, excessive posterior retraction of the SCM is required to expose the lesion. This procedure dramatically increases the risk of traction injury to the accessory nerve, and postoperative complications such as trapezius dysfunction and shoulder pain may occur, affecting the patient's quality of life. The PG and SCM are both enclosed within the investing fascia and have a close anatomical relationship (17). In the classic approach, during the process of retracting the skin flap to expose the anterior edge of the SCM, the PG is easily injured due to unclear layer separation, which may lead to postoperative parotid fistula and facial nerve injury, affecting the patient's appearance and salivary secretion function (24).

Given the above limitations, to further improve the safety and practicality of the ELA and promote its wider clinical application, our team aimed to “avoid injury and optimize exposure” and modified the classic approach based on the anatomical characteristics of the PVF, proposing a new scheme for exposing the UHRCS from the posterior side. This scheme has been repeatedly verified in anatomical training and clinical application.

### 4.4 Design, technical key points, and advantages of the novel technique

#### 4.4.1 Design concept and core anatomical basis of the technique

The core design of this technique is to fully expose the AOJ after standardized positional design, making full use of the anatomical association between the PVF and the UHRCS. Additionally, it constructs a new posterior corridor for lesion exposure, thereby avoiding the limitations of the classic ELA. This posterior corridor is anatomically rooted in the “post-carotid space” as defined (25), who systematically characterized the C1-C2 anterolateral corridors and identified the space between the carotid sheath and the PVF as the safest route for ventral access. Our technique extends this anatomical concept cranially to the skull base exit of the jugular bulb, effectively creating a continuous surgical channel from C2 to the JB that avoids direct manipulation of the carotid vasculature. This anatomical characteristic provides a natural anatomical space for exposing the UHRCS from the posterior side through the PVF, serving as the core anatomical basis and novelty of this technique.

#### 4.4.2 Technical key points of the novel concept

Standardized positional adjustment is a key step in this technique, with the core goal of “fully opening the AOJ and expanding the surgical corridor.” Specifically, based on the lateral decubitus position, it is combined with the “three-action coordination” of the head—rotating the head 30∼45° to the contralateral side, fully retracting the chest (the distance between the chin and the sternal manubrium is 1∼2 transverse fingers), and lowering the top of the head by approximately 20°—to place the MT at the highest point. This adjustment not only expands the posterior surgical corridor between the MT, OC, and C1TP but also displaces the CS to the posteroinferior side of the SCM via cervical rotation, bringing the lesion closer to the surgical corridor and reducing exposure difficulty. This standardized positional adjustment is precisely tailored to the anatomical range of the UHRCS, which maximizes the exposure efficiency of the UHRCS region and aligns with the surgical criteria for UHRCS exposure, further reflecting the guiding role of UHRCS anatomical and surgical classification in technical design. Anesthetic operations must be tailored to the characteristics of the position and surgical approach, with specific requirements including: avoiding IJV puncture (to prevent increased venous pressure or thrombosis), prioritizing subclavian vein puncture or preoperative placement of a PICC (Peripherally Inserted Central Catheter); adopting nasotracheal intubation to prevent obstruction of the surgical field by the mandibular angle after mouth opening, thereby further optimizing the operating space.

The exposure operation of the ELA based on the anatomical principles of the PVF better respects the natural spaces of anatomical structures and the pathophysiological characteristics of lesions involving the UHRCS. Its key operating points are summarized as follows: 1. Exposing the PVF: Reflect the SCM anteriorly to expose the PVF; 2. Expanding the operating space: If the atlas transverse process significantly obstructs the surgical field, first displace the V3 segment of the VA (to avoid vascular injury), then grind away the atlas transverse process to further expand the exposure range. In adhering to this principle, our method is consistent with the classic concept (26), who emphasized that extensive drilling of the occipital condyle and C1 lateral mass should be avoided unless necessitated by tumor invasion. Consistent with their findings, our technique employs a “minimal invasive drilling” strategy, limiting bone removal to only what is required for corridor access, thus preserving craniocervical stability. 3. Exposing the lesion (constructing a more reasonable surgical corridor): Incise the PVF along the posterior surgical corridor to directly expose the UHRCS and the lesion, eliminating the need for IJV dissection and simplifying the surgical procedure.

#### 4.4.3 Significant advantages of this technique

##### 4.4.3.1 Lesion exposure and neurovascular protection

In terms of lesion exposure, the standardized posture adjustment of the ELA based on the anatomical principles of the PVF rotates the CS to the posterior side of the SCM via neck rotation, enabling easier exposure of the UHRCS. Meanwhile, retracting the skin flap and SCM together anteriorly not only improves operational efficiency by making full use of the PVF's anatomical principles but also retracts the subcutaneous PG anteriorly with minimal injury (17). Additionally, this method of exposing the UHRCS by incising the PVF through the posterior surgical channel fully utilizes the PVF's anatomical characteristics, avoiding traction on the accessory nerve, damage to the PG, and transposition of the IJV.

##### 4.4.3.2 In terms of EJV protection

Considering that when there is a space-occupying lesion in the UHRCS, the IJV is often narrowed or occluded due to compression, and intracranial venous reflux is usually taken over by the EJV (which is frequently compensatorily dilated). Injuring such compensatory veins with normal drainage function may affect intracranial venous reflux. This technique reduces this risk through two designs: first, the skin flap design retracts the skin and SCM together anteriorly, eliminating the need to separate them; second, the incision only needs to reach the posterior edge of the SCM, allowing for a smaller and more posterior skin flap design, further reducing the risk of EJV injury.

##### 4.3.3.3 Making full use of anatomical principles and lesion characteristics

The neurovascular structures in the UHRCS exhibit a characteristic arrangement: the IJV is located at the outermost side, cranial nerves (mainly the vagus nerve and hypoglossal nerve branches) are in the middle, and the ICA is at the anteromedial side. First, based on this anatomical rule, space-occupying lesions of neural origin (e.g., schwannomas) often push the IJV laterally as they grow. Mainly when the IJV remains patent and cannot be ligated due to functional needs, surgical exposure through the posterior PVF space has significant advantages: this pathway can fully utilize the natural anatomical space between the lesion and the IJV, leveraging the “surgical channel” formed by tumor growth to effectively eliminate the obstruction of the IJV to the surgical field while reducing traction on the IJV and lowering the risk of vascular injury. Therefore, when using the ELA, UHRCS lesions can be directly exposed by incising the PVF through the posterior operation channel, eliminating the need for IJV dissection (as required in the classic approach), thereby greatly simplifying the surgical process and significantly improving surgical efficiency.

##### 4.4.3.4 Combined operations for individualized treatment

Furthermore, this technique has good surgical compatibility and can be flexibly combined with other targeted operations according to the growth rule and the lesion's range to achieve precise, individualized treatment. For example, if the lesion communicates with the JB, a mastoidectomy can be combined; if the lesion communicates with the subdural space, the OS needs to be opened; if the lesion communicates with the HC, the HC needs to be drilled (Case 2, Figure 9). Therefore, the minor technical refinement to the ELA described in this study is more like a “source code” for managing CCJ lesions. In clinical practice, it is necessary to conduct a case-specific analysis to achieve “tailored treatment” based on specific conditions. This not only avoids excessive “unnecessary exposure” but also ensures thorough lesion management, further improving the treatment effect of complex lesions.

### 4.5 Compared with the classic anterolateral approach

#### 4.5.1 Differences in core design concepts

The core of Bernard George's classic anterolateral approach is “dissection along or within the anterior border of the sternocleidomastoid muscle (SCM)” to expose the neurovascular structures in the anterolateral cervical region, with its design focus on solving the exposure problem of the ventral foramen magnum and the lower cranial nerve region (18, 27). In contrast, the core innovation of our study is “constructing a posterior surgical corridor based on the natural anatomical space of the PVF”. Instead of dissecting along or within the anterior border of the SCM, we retract the SCM anteriorly as a whole and use the natural space between the PVF and the carotid sheath (CS) to expose the UHRCS. The design concept focuses on the safe exposure of the UHRCS, a unique anatomical region, which is essentially different from the design goals and anatomical paths of the classic anterolateral approach.

This distinction is further highlighted by comparison with the contemporary juxtacondylar approach (28), which combines anterolateral cervical dissection with limited mastoidectomy for jugular foramen tumors. While their approach effectively addresses lesions with extensive cervical extension requiring facial nerve mobilization, it necessitates posterior retraction of the SCM and direct manipulation of the cervical neurovascular bundle. Our PVF-based ELA, by contrast, is uniquely optimized for the UHRCS segment at the skull base-C2 junction, achieving exposure through a posterior corridor that obviates the need for such extensive anterolateral dissection.

#### 4.5.2 Differences in anatomical localization and exposure range

The exposure range of the classic anterolateral approach mainly covers the ventral foramen magnum, lower cranial nerves, and vertebral artery, extending to the upper third of the parapharyngeal space (29, 30), but it is not specifically designed for the UHRCS, a special segment of the carotid sheath at the skull base-high cervical junction (C1-C2 level). In contrast, our modified ELA is specifically designed for UHRCS-involving lesions. Through standardized head position adjustment (mastoid tip elevation, cervical flexion and rotation), it focuses on optimizing the triangular corridor formed by the mastoid tip, C1 transverse process, and occipital condyle, which can accurately expose the UHRCS and the involved jugular bulb and hypoglossal canal regions, making up for the deficiency of the classic anterolateral approach in exposing the UHRCS region.

#### 4.5.3 Differences in clinical indications

The classic anterolateral approach is mainly applicable to foramen magnum meningiomas, lower cranial nerve schwannomas (not explicitly involving the UHRCS), and vertebral artery diseases (18, 27, 29–31). In contrast, our PVF-based ELA is specifically targeted at UHRCS-involving lesions—these lesions are located at the skull base-high cervical junction, with the anatomical complexity of both skull base and high cervical lesions. The classic anterolateral approach is difficult to achieve precise and safe exposure, while our approach can effectively avoid the risk of neurovascular injury, serving as a supplement and extension to the indications of the classic anterolateral approach.

### 4.6 Postoperative comorbidity of the proposed surgical technique

In terms of postoperative complications, two cases developed subcutaneous effusion, which achieved complete recovery after one week of pressure bandaging intervention, and no new neurological complications were observed in the remaining cases. Meanwhile, we explicitly acknowledge the inherent limitation that comorbidity may be underestimated in skull base surgery (15, 32), which is a common challenge in clinical studies involving this complex surgical field. Existing studies have only reported the effects of classic approaches in the treatment of extracranial jugular foramen schwannomas, but have not involved special cases with UHRCS involvement (33); all 11 cases in our study explicitly involve the UHRCS, and complete resection was achieved with the PVF-based modified ELA without severe complications, which supplements the treatment experience of such special lesions and further improves the surgical treatment system for jugular foramen region lesions.

The balance between surgical benefits and comorbidity risks also merits emphasis in the context of patient informed consent. The surgical technique proposed in this study demonstrates a more favorable risk-benefit ratio for lesions involving the UHRCS, primarily by mitigating the risks of neurovascular injury (i.e., IJV and accessory nerve injury) and salivary gland dysfunction. All potential advantages and corresponding risks associated with the proposed technique were fully disclosed to patients during the informed consent process, so as to guarantee their right to make informed and autonomous clinical decisions.

### 4.7 Limitations of the proposed surgical technique

#### 4.7.1 Surgical trajectory

The posterior surgical corridor may limit visualization of anteriorly located lesions in the parapharyngeal space, requiring careful preoperative imaging evaluation to select appropriate candidates.

#### 4.7.2 Mediolateral exposure

The mediolateral exposure range is slightly narrower than that of the classic ELA, which may pose challenges for large UHRCS-involving lesions; we recommend combined approaches for such cases.

#### 4.7.3 Atlas drilling and V3 vertebral artery transposition

Atlas drilling carries a low risk of vertebral artery injury, and V3 transposition (when needed) may increase the risk of temporary neurological symptoms; we emphasize the need for meticulous anatomical identification and standardized drilling techniques to mitigate these risks.

In summary, through an in-depth exploration of anatomical principles and innovative research on surgical approaches, this study provides a safer, more efficient treatment scheme for complex UHRCS lesions. It not only addresses the limitations of the classic approach but also offers new ideas for the precise and individualized development of CCJ surgical techniques. However, this study has certain limitations. The primary task in the future is to expand the clinical sample size to fully verify the long-term efficacy and universality of this technique. Additionally, systematic technical training can be implemented to address challenges in technology promotion, and subgroup analyses can be conducted for lesions of different pathological types to further expand the clinical indications and promote standardized application of this technology in more complex skull base lesions.

## 5 Conclusion

The PVF anatomical principle-based ELA effectively avoids the risks of the classic ELA. It reduces the learning curve by standardizing the surgical position, optimizing the SCM retraction direction, and constructing a surgical corridor using key anatomical landmarks. It provides a safer, more efficient surgical option for treating UHRCS-involved lesions.

## Data Availability

The original contributions presented in the study are included in the article/Supplementary Material, further inquiries can be directed to the corresponding author.
